# Microbiome dysbiosis occurred in hypertrophic scars is dominated by *S. aureus* colonization

**DOI:** 10.3389/fimmu.2023.1227024

**Published:** 2023-08-23

**Authors:** Jiarong Yu, Zhigang Mao, Zengding Zhou, Bo Yuan, Xiqiao Wang

**Affiliations:** ^1^ The Department of Burn, Ruijin Hospital, Shanghai Jiaotong University School of Medicine, Shanghai, China; ^2^ The Department of Plastic Surgery, Ninth People's Hospital, Shanghai Jiaotong University School of Medicine, Shanghai, China

**Keywords:** microbiome dysbiosis, *S. aureus*, Vancouver Scar Score, hypertrophic scar, inflammation

## Abstract

**Background:**

The mechanisms of hypertrophic scar formation and its tissue inflammation remain unknown.

**Methods:**

We collected 33 hypertrophic scar (HS) and 36 normal skin (NS) tissues, and detected the tissue inflammation and bacteria using HE staining, Gram staining, and transmission electronic microscopy (TEM), *in situ* hybridization and immunohistochemistry for MCP-1, TNF-α, IL-6 and IL-8. In addition, the samples were assayed by 16S rRNA sequencing to investigate the microbiota diversity in HS, and the correlation between the microbiota and the indices of Vancouver Scar Scale(VSS)score.

**Results:**

HE staining showed that a dramatically increased number of inflammatory cells accumulated in HS compared with NS, and an enhanced number of bacteria colonies was found in HS by Gram staining, even individual bacteria could be clearly observed by TEM. *In situ* hybridization demonstrated that the bacteria and inflammation cells co-localized in the HS tissues, and immunohistochemistry indicated the expression of MCP-1, TNF-α, IL-6, and IL-8 were significantly upregulated in HS than that in NS. In addition, there was a significantly different microbiota composition between HS and NS. At the phylum level, *Firmicutes* was significantly higher in HS than NS. At the genus level, *S. aureus* was the dominant species, which was significantly higher in HS than NS, and was strongly correlated with VSS indices.

**Conclusion:**

Microbiome dysbiosis, dominated by *S. aureus*, occurred in HS formation, which is correlated with chronic inflammation and scar formation, targeting the microbiome dysbiosis is perhaps a supplementary way for future scar management.

## Introduction

Human hypertrophic scars (HS), often occurring as a result of burns, trauma, or surgery, are a major clinical problem affecting over 80 million people worldwide each year (1). It appears at 1-2 months after wound healing, then develops hyperplasia within 6-24 months, which is accompanied by redness, elevation, itching, and pain. HS often causes severe cosmetic, functional, and even psychiatric impairment, greatly reducing the life quality (2). Many factors that contribute to hypertrophic scars have been reported, including delayed healing (3, 4), hypoxia microenvironment (5, 6), mechanical force, aberrant gene expression (7, 8), etc. Our understanding of HS is improving. However, these mechanisms cannot fully explain the definitive processes of scar hyperplasia, and the relevant therapies do not achieve a satisfactory effect on scar formation. Therefore, exploring the “culprit” of HS formation is of great importance.

Clinically, human adult HS was also regarded as a chronic inflammatory disease as persistent inflammation has been detected during HS formation (9). In contrast, there is no scar formation after wound healing in the fetus due to the lack of inflammatory responses in the fetus (10, 11). Thus, chronic inflammation is perhaps a “marker” that determines scar formation. Based on these observations, Ogawa and Akaishi hypothesized that HS and keloid are the same disorder depending on the extent of inflammation, and they classified keloid and HS as strong and moderate inflammation-induced scars, respectively (12). However, the factors that cause chronic inflammation in scar tissue have not been elucidated.

Currently, it is becoming increasingly evident that tissue inflammation is correlated with microbiome dysbiosis in many diseases (13, 14). Microbiota dysbiosis produces diverse metabolites that alter microbiome metabolism and host metabolism, affect adaptive immunity, and trigger inflammation (15, 16). Indeed, the skin microbiome is an ecosystem comprising commensal bacteria that not only reside in the skin surface (17), but also extend to subepidermal compartments including the skin dermis and adipose tissues (18). The pathogen invasion causes microbiota dysbiosis and inflammation. For example, Hidradenitis suppurativa is a chronic inflammatory skin disease of the hair follicle. A previous study revealed that *Propionibacterium* was the causing pathogen to induce microbiome dysbiosis (19). Atopic dermatitis (AD) is also a chronic inflammatory disease, and *S. aureus* is highly prevalent in AD skin (20, 21).

Therefore, in this study, we would like to know whether the microbiota dysbiosis occurred in scar tissues, and which bacteria dominated in the HS.

## Materials and methods

### Collection of HS and NS samples

The study was performed at the Burn Department, Ruijin Hospital, Shanghai Jiao Tong University School of Medicine, from February to December, 2021, and was approved by the Ethics Committee of Ruijin Hospital, Shanghai Jiao Tong University School of Medicine. All participants provided written informed consent.

Patients (2–61 years of age, male 19 and female 14) with burn-related HS, located on the limbs or trunks, which were featured by elevation, redness, and hardness, and needed scar excision and skin transplantation, were enrolled in this study. Meanwhile, 36 normal skin (NS) samples were harvested from these patients or other patients after skin transplantation, and all the donor sites were abdomen.

### Inclusion and exclusion criteria

The patients with scar duration ranging from 6 to 24 months were included, none of the participants received any antibiotics (systemic or oral therapy) within recent 3 months, and patients with keloids, scar ulcers, diabetes, or cardiovascular disease were excluded.

### Perform Vancouver Scar Scale score before surgery

Before surgery, the scars were assessed by Vancouver Scar Scale (VSS) score, and the indices of pigment, thickness, pliability, itching/pain, and vessel were assessed for each sample.

### Part 1: tissue processing for histochemistry experiment

During the surgery, the process was strictly disinfected to avoid bacterial contamination.

After surgery, the tissue was quickly put into the sterile specimen box, which had been irradiated under a UV light for 90 min, then tissue samples were further irradiated with UV light for 30 min, and subsequently washed 3 times with sterile PBS.

The samples were then fixed in 10% buffered formalin, embedded in paraffin blocks, and cut into 6-μm-thick sections. The sections were subjected to hematoxylin and eosin (H&E) staining, Gram staining, electron microscopy, *in situ* hybridization, and immunohistochemistry.

### H&E staining for inflammation cells

H&E staining was performed according to a standard protocol. Briefly, after deparaffinization and rehydration, sections were stained with hematoxylin solution for 5 min followed by 5 dips in 1% acid ethanol (1% HCl in 70% ethanol) and then rinsed in distilled water. Then the sections were stained with eosin solution for 3 min and followed by dehydration with graded alcohol and clearing in xylene. The mounted slides were then examined and photographed using an Olympus BX53 fluorescence microscope (Tokyo, Japan). The experiment was repeated three times, the number of inflammation cells was calculated per view, and 8 samples were tested.

### Gram staining for bacteria detection

Gram staining was performed according to the protocol of the manufacturer (Beijing Solarbio Science& Technology, Beijing, China). Briefly, the heat-fixed smears on slides were flooded with 0.2% Victoria blue for 30 seconds and washed with tap water, smears were decolorized with 2% picric acid ethanol, and cells were counterstained with 0.004% fuchsin for 30 seconds and then washed with tap water. The positive staining was blue. The experiment was repeated three times, the area of gram-positive staining was calculated by Image J software, and 8 samples were tested.

### Transmission Electron microscopy for bacteria

Transmission Electron microscopy (TEM) was performed as previously described (22). First, tissue samples were fixed with 2.5% glutaraldehyde followed by 1% OsO4. Then, the samples underwent serial dehydration, soaking, embedding in epoxy resin, and sectioning into ultra-thin 60-nm sections. The sections were stained with a solution of uranyl acetate and lead citrate, and then a transmission electron microscope (HITACH 500, Hitachi, Ltd., Tokyo, Japan) was used at a voltage of 75 kV to observe the bacteria within the scar tissues. The experiment was repeated three times, the number of bacteria was calculated per view, and 5 samples were tested.

### *In situ* hybridization for tissue bacteria and inflammation cells

For fluorescence *in situ* hybridization, oligonucleotide probes (EUB338: 5′-GCTGCCTCCCGTAGGAGT-3′) conjugated with Alexa Flour 488 (Invitrogen, Carlsbad, CA, USA), targeting 16S rRNA, were used to label bacteria, and CD11b antibody conjugated with Alexa flour 594 (Invitrogen) was used to label the inflammatory cells. Briefly, sections were immediately fixed with RNase-free 4% formaldehyde and permeabilized, followed by dehydration with ethanol (50%, 75%, 100%). The sections were hybridized with EUB338 (5 ng/mL) overnight at 37°C, and the excess and non-specifically bound probe was washed with phosphate-buffered saline (PBS)×2. Then, sections were and incubated with rabbit anti-human CD11b primary antibody overnight at 4°C, washed with PBS×2, and incubated with Alexa Fluor 594-conjugated donkey anti-rabbit antibody at room temperature. Then the nuclei were stained with 4′,6-diamidino-2-phenylindole (DAPI; H-1200; Vector Laboratories, Burlingame, CA, USA). Images were captured using a con-focal laser scanning microscope (Carl Zeiss, Oberkochen, Germany). The experiment was repeated three times, the area of positive staining was calculated by Image J software, and 8 samples were tested.

### Immunohistochemistry for inflammation cytokines MCP-1, IL-6, IL-8, and TNF-α

VECTASTAIN Elite Avidin-Biotin Complex Kit (Maixin Biotech. Co, Fuzhou, China) was used for immunohistochemical staining of MCP-1, TNF-α, IL-6, and IL-8 according to the manufacturer’s instruction. The primary and secondary antibodies used are listed in **Table 1**. Briefly, sections of the paraffin-embedded femurs were kept at 60°C for 24 h in the oven and then followed by deparaffinizing with xylene and hydrating with an ethanol gradient (100%–70%). After successively incubating with antigen retrieval solution (Shanghai Shunbai Biotechnology Company; Shanghai, China) and 3% H_2_O_2_ for 30 min, the slides were rinsed with water and incubated with the primary antibody MCP-1, IL-6, IL-8, and TNF-α overnight at 4°C(1:100 dilution; Santa Cruz Biotechnology Inc, Santa Cruz, CA). The next day, the slides were rinsed and incubated with the corresponding secondary antibody (1:100 dilution; Santa Cruz Biotechnology Inc)) for 30 min followed by 3,3′-diaminobenzidine (DAB) and hematoxylin staining, respectively. The slides were then examined and photographed using an Olympus BX53 fluorescence microscope (Tokyo, Japan). The experiment was repeated three times, the area of positive staining was calculated by Image J software, and 8 samples were tested.

**Table 1:** Background information of participants.

	Items	NS	HS	P value
**Basic information**	Age(years)	18.61 ± 3.01	19.67 ± 3.40	0.82
	Sample No.	36	33	-
	Male No.	19	19	0.69
	Female No.	17	14	
	Scar duration (months)	-	10.82 ± 0.96	-
**VSS scores**	Pigment	0	2.15 ± 0.36	<0.0001
	Thickness	0	2.52 ± 0.51	
	Pliability	0	2.82 ± 0.58	
	Itching/pain	0	1.03 ± 0.53	
	Vessel	0	1.48 ± 0.67	

### Part 2: tissue preparation for 16S-rRNA sequencing

As mentioned above, after the wash of the tissues, the epidermis of HS and NS were removed using a scalpel. Then the samples were stored at -80°C before genomic RNA extraction.

### 16S-rRNA sequencing

Tissue genomic DNA was extracted from 0.1 g frozen skin and scar samples using MP Fast DNA SPIN Kit for Soil according to the manufacturer’s protocol (23). Briefly, the DNA concentration and purification were measured, and the DNA quality was detected by electrophoresis. The 16S-rDNA gene were amplified using primers 341F: 5′-ACTCCTACGGGRSGCAGCAG-3′, and 806R: 5′-GGACTACVV GGGTATCTAATC-3′. PCR was performed. The reactions were performed on a thermocycler PCR system (GeneAmp 9700, ABI, USA). All PCR products were purified using an AxyPrep DNA Gel Extraction Kit (Axygen Biosciences, Union City, CA, USA) and quantified using QuantiFluor™-ST (Promega, USA). Purified and pooled amplicon libraries were paired-end sequenced (2×300) on the Illumina MiSeq platform (Illumina, San Diego, USA) according to the standard protocols by Majorbio Bio-Pharm Technology Co., Ltd. (Shanghai, China).

Raw sequence reads were demultiplexed, quality-filtered, merged, and clustered into OTUs with a 97% similarity cutoff using UPARSE (version 7.1, http://drive5.com/uparse/), and chimeric sequences were identified and removed using UCHIME. The taxonomy of the acquired OTUs was analyzed using the RDP Classifier Bayesian algorithm (http://rdp.cme.msu.edu/) against the SILVA database (version128) with a confidence threshold of 70%.

### Statistical analysis

Data generated from H&E staining, Gram staining, Masson staining, TEM, *in situ* hybridization, and immunohistochemistry analyzed by Image J software, and the data was expressed as mean ± SD, and two groups were red by two-tailed Student’s t-test.

For the 16S sequencing data, all statistical analyses were performed using R packages (V·2·15·3) as follows.

Microbiota α-diversity, which represents microbial diversity within an individual group, was computed in QIIME through the whole tree phylogenetic diversity metric. And Shannon index was used.

Microbiota β-diversity, which indicates inter-variability of microbial diversity between groups, was examined through weighted UniFrac distances in QIIME and hierarchical clustering based on the unweighted pair group Method, and principal coordinates analysis (PCOA) was used.

To test the difference in microbiome composition between the two groups, the Kruskal-Wallis test was used, and phylum, genus, and species levels were selected for this analysis.

To evaluate the correlation between microbiota and clinical variables, the relationship was calculated through Spearman correlation.

For all statistical analyses, a 2-sided P <.05 was accepted as statistically significant.

## Results

### Patient characteristics

**Figure 1:**
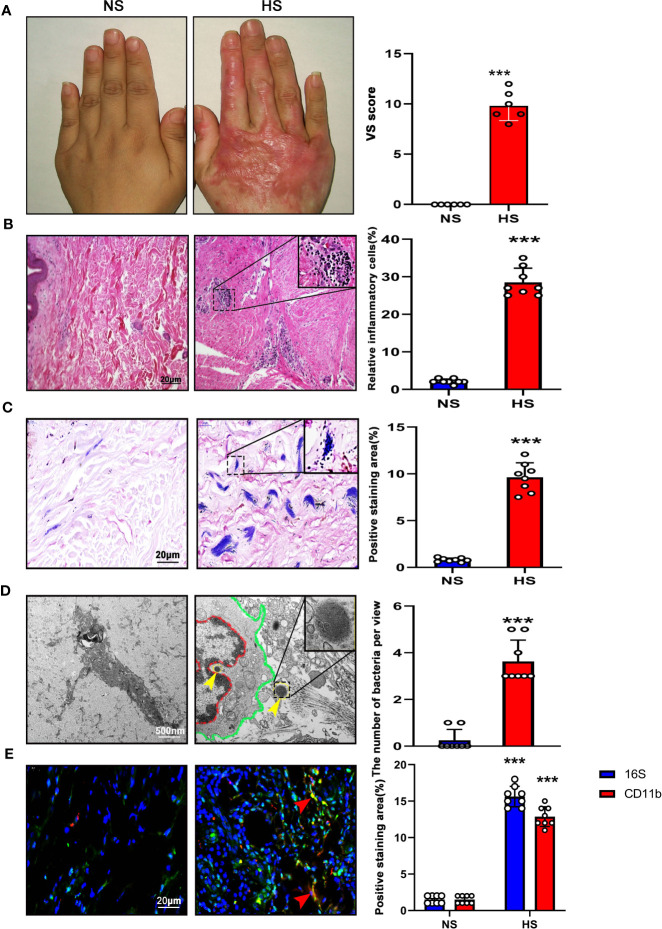
High bacterial content and inflammation in HS tissues. **(A)** Representative image of NS (left) and HS(right). HS appears red and has elevated shape compared to the NS. **(B)** H&E staining of tissue sections from NS and HS. Areas enclosed by the black box are magnified and shown in the top right corner. Scale bar: 20μm.The images are representative of three experiments with similar results. Data are shown as mean ± SD. ***P<0.001. **(C)** Gram staining of tissue sections from NS and HS. Scale bar: 20μm.The images are representative of three experiments with similar results. The area of gram positive staining was calculated by image J and shown as mean ± SD. ***P<0.001. **(D)** High-resolution transmission electron microscopy (HR-TEM) image of NS and HS tissue section. Macrophages (red line and green line) were engulfing bacteria(yellow arrow). A bacterium enclosed by the black box is magnified and shown in the top right corner. Scale bar: 500 nm. The number of bacteria was calculated by image J and shown as mean ± SD. ***P<0.001. **(E) $ **Representative immunofluorent double-staining of NS and HS tissue. Staining: CD11b (green), inflammatory cell marker; 16s (red), bacteria marker; DAPI (blue). Scale bar: 20μm. The images are representative of three experiments with similar results. The area of positive staining was calculated by image J and shown as mean ± SD. ***P<0.001.

In our study, 33 HS (male 22, female 11) and 36 NS (male 24, female 12) were involved in this study, and average ages were 19.67 ± 3.50 (HS) and 18.61 ± 3.27 (NS) respectively, mainly located at the neck, trunk, and upper and lower extremity. There was no significant difference between the 2 groups in sex (p=0.83), age (p=0.20), and sample location (p=0.92), but the indices of VSS score were significantly higher in HS than that in NS (**Figure 1A**; **Table 1**).

### Higher inflammation and bacteria count in HS

H&E staining showed almost no inflammatory cells in the NS with loose collagen arrangement, however, in HS tissue, many clusters of inflammatory cells with dense collagen fibers were detected (**Figure 1B**).

According to Gram staining, few bacteria colonized in the sub-epidermis of NS tissue, instead, a large number of gram-positive bacteria colonizing in HS tissue was observed. (**Figure 1C**).

Using TEM, we confirmed the presence of bacteria in HS tissues, whereas almost no signs of bacteria were detected in NS dermis. The individual bacteria in HS tissues appeared round or elliptical with wave-like membranes. Interestingly, it was found that a bacterium was swallowed by an inflammatory cell (**Figure 1D**).

In addition, 16S-rRNA and CD11b staining were used to label the bacteria and inflammation cells respectively. According to the result, the bacteria clusters could be seen in HS with inflammatory cells distributed around, both were increased significantly in HS than that in NS (**Figure 1E**).

Immunohistochemistry revealed that NS tissues showed extremely low expression of MCP-1, IL-6, IL-8, and TNF-α. In contrast, their expression level was significantly higher in HS(**Supplement Figure**).

These results demonstrated that higher bacteria count and tissue inflammation were present in HS.

### α-diversity and β-diversity between two groups

The 16S rRNA amplicon sequencing analysis was used to sequence the microbiome in 36 NS and 33 HS samples. In total, 11,222 OTUs, 1 domain, 1 Kingdom, 56 Phyla, 134 Classes, 370 Orders, 691 Families, 1,652 Genera, and 3,417 Species were obtained from all samples.

**Figure 2:**
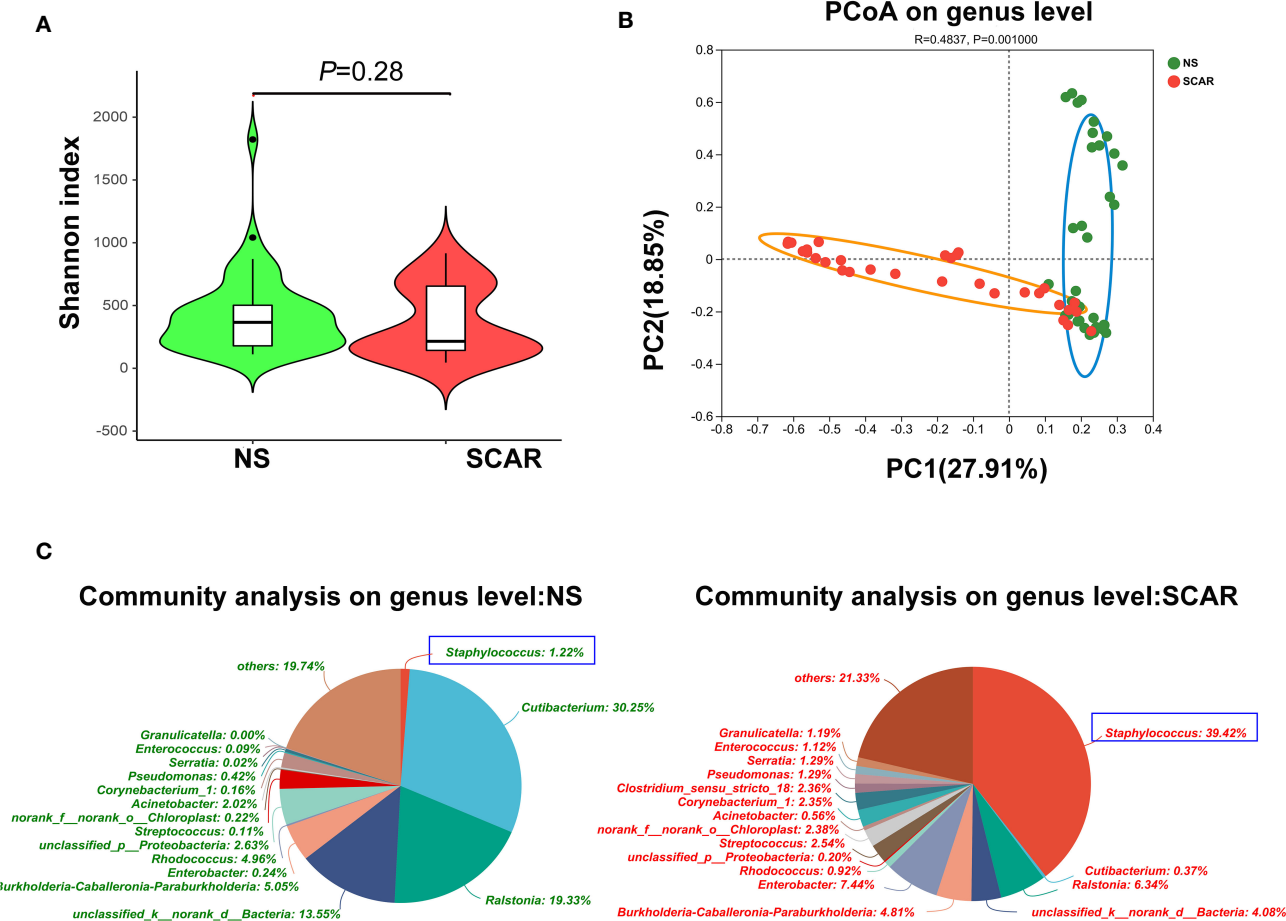
α-diversity and β-diversity of bacterial community in HS and NS. **(A)** α-diversity comparison with Shannon index showed there was no difference between HS and NS (*P=*0.210) **(B)** β-diversity comparison with PCoA showed a clear separation between HS and NS (R=0.4970, *P=*0.001). **(C)** The pie chart showed the bacteria community composition at genus level in two groups.

In order to compare the microbiota richness and evenness between HS and NS, the a-diversity was calculated using the Chao, Shannon, and Simpson index, however, no significant difference was found between the two groups (**Figure 2A**). (Chao index p=0·103, Shannon index p=0·28, and Simpson index p=0·176, respectively)

β-diversity reflects the intra-group variability of the microbiome composition between two groups. When the indices of PCoA were used, the results showed that there was a clear separation between HS and NS (R=0·4970, *P*=0·001, **Figure 2B**), indicating a significantly different microbiota composition pattern between scar and NS.

### Microbiome dysbiosis occurred in HS

The community abundance of each sample was counted at different taxonomic levels with R package, and the microbiome compositions of both groups were visualized by pie chart (**Figure 2C**). Then the microbiota differences between NS and HS were compared respectively at phylum, genus, and species levels.

**Figure 3:**
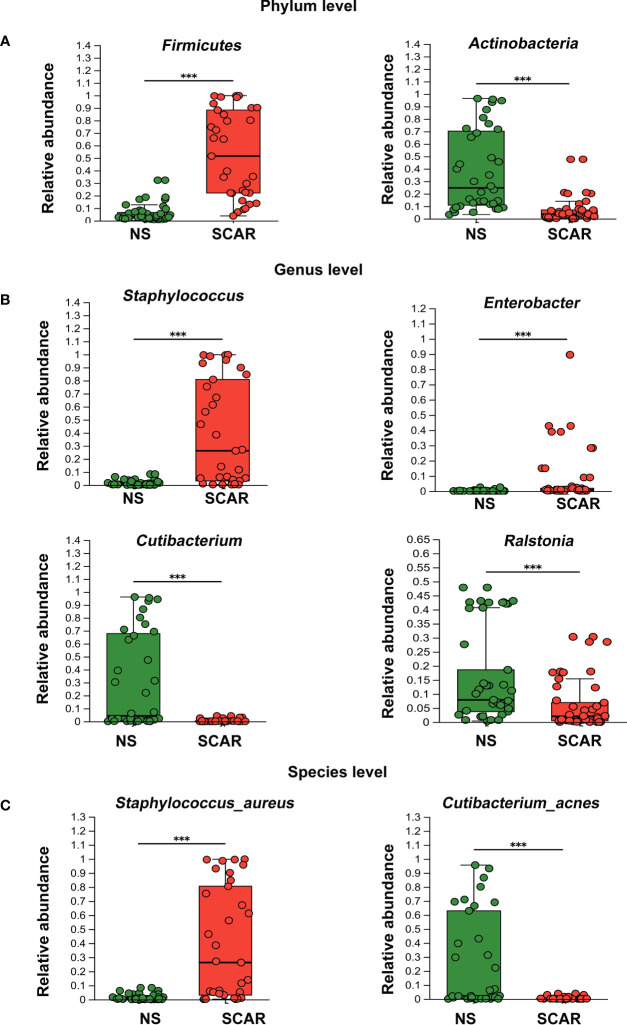
Different levels of microbiota abundance between HS and NS. **(A)** On Phylum level, the major difference of microbiota abundance between two groups. ***P<0.001 **(B)** On Genus level, the major difference of microbiota abundance between two groups. ***P<0.001 **(C)** On Species level, the major difference of microbiota abundance between two groups(the unclassified species were not listed). ***P<0.001.

At the phylum level, *Actinobacteria, Firmicute, Proteobacteria, and Bacteroidetes* were the major microbiota in HS and NS tissues, but the proportion varied. The *Firmicute* was significantly higher in HS than that in NS (53·2% & 5·52%, *P*=7·18×e^-11^), whereas *Actinobacteria* in HS was significantly lower than NS (9·19% & 38·21%, *P*=1·735×e^-7^). And the abundance of *Proteobacteria* and *Bacteroidetes* did not significantly differ between the two groups (data not shown in figure, *P*=0·065, *P*=0·59, respectively) (**Figure 3A**).

At the genus level, NS mainly harbored *Cutibacterium* (30·25%), *Ralstonia* (19·33%), *Rhodococcus* (4.96%), etc. Of note, HS tissues exhibited different proportions of the microbiome, named *Staphylococcus* (39·42%), *Enterobacter* (7·44%), *Ralstonia* (6·34%), *Streptococcus* (2·54%), etc. The abundances of *Staphylococcus*, *Enterobacter, and Streptococcus* in HS were significantly higher than that in the NS (39·42% & 1.22%, P=9·31×e-8; 7·44% & 0.24%, P=0·000006; 2·54% & 0.11%, P=0·0007 respectively). However, the prevalence of *Cutibacterium* and *Ralstonia* were significantly lower in HS tissues than that in NS (0.37% & 30·25%, P=1·14×e-7; 6.34% & 19·33%, P=0·0005 respectively) (**Figure 3B**).

At the species level, *S. aureus* had the highest prevalence in scar tissues, which was significantly greater than that in NS tissues (39·71% & 1·88%; *P*=1·13×e^-7^). Secondly, unidentified *Enterobacter* and unclassified *Streptococcus* species were also significantly more prevalent in scar tissues than that in NS (7·44% & 0.22%, *P*=8·3×e^-5^; 2·54% & 0.09%, *P*=3·42×e^-4^, respectively), but the proportion was not high (data not shown in figure). However, *Cutibacterium acnes*, which was dominant in NS tissue, were significantly reduced in HS (28·89% & 0·33%, *P*=4·41×e^-8^) (**Figure 3C**).

Normally, 16s sequencing could not identify species, but in our study, *S.aureus* and *Cutibacterium acnes* were identified. The above results indicate that microbiome dysbiosis occurred in HS, which was dominated by *S.aureus*.

### Genus *Staphylococcus, Enterobacter*, and *Streptococcus* are positively correlated with scar formation

**Figure 4:**
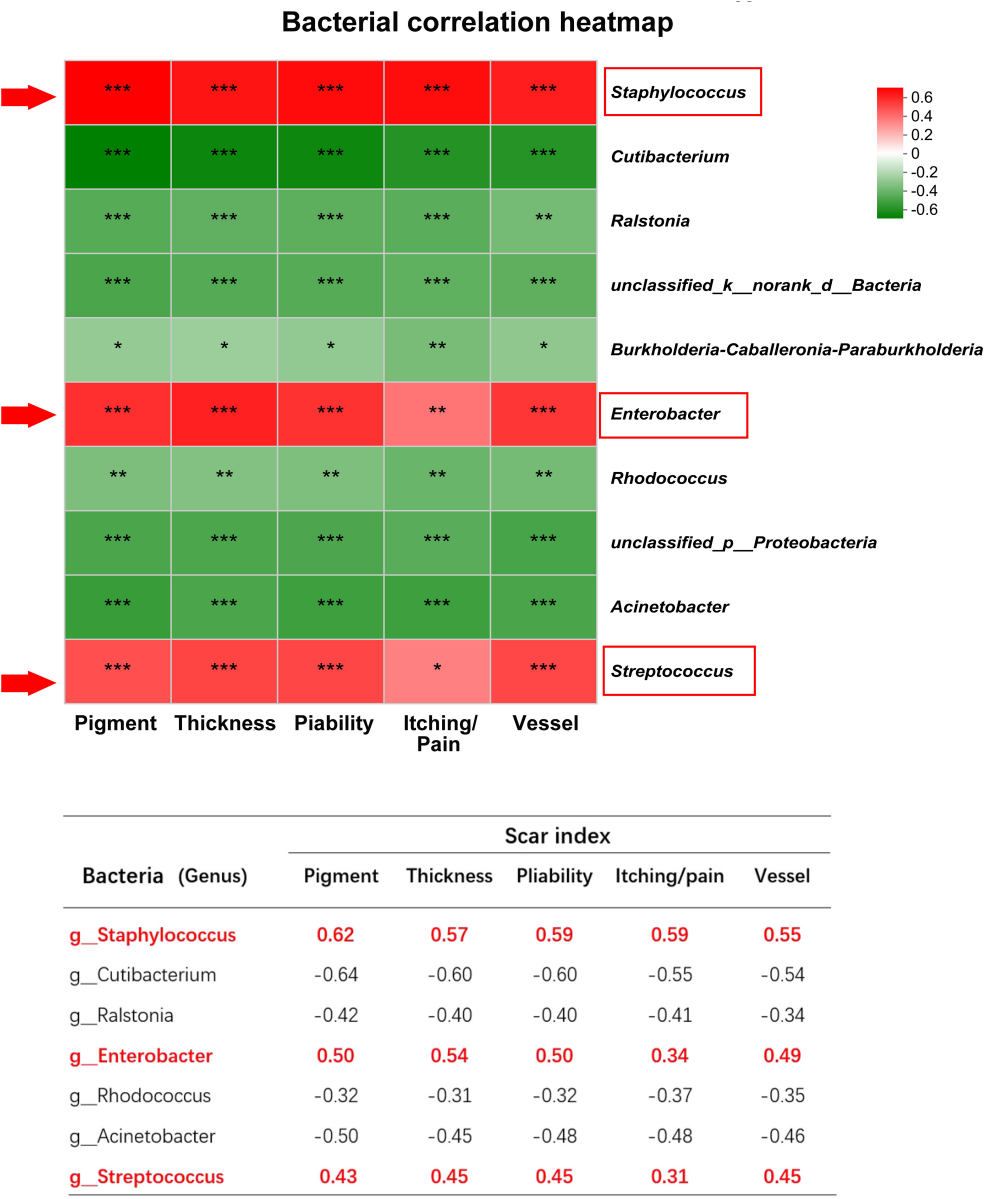
The correlation between microbiota and scar indices. *** indicates P<0.001, ** indicates P<0.01, * indicates P<0.05.

In order to assess the correlation between the bacteria and the scar, the VSS indices including pigment, thickness, pliability, itching/pain, and vessel were used to evaluate HS clinical severity. For each HS patient, we calculated the Spearman correlation between the VSS scores and individual bacterial abundance, represented by a heatmap plot. Red indicates a positive correlation between bacterial content and clinical severity, and green represents a negative correlation. The results revealed that genus *Staphylococcus, Enterobacter*, and *Streptococcus* were positively correlated with clinical indices (**Figure 4**). However, genus *Cutibacterium, Ralstonia*, etc. were negatively correlated with scar indices. For instance, g_*Staphylococcus* had a strong correlation with chroma (r=0.6230, p<0.001), vessel (r=0.5513, p<0.001), thickness (r=0.5743, p<0.001), pliability (r=0.5965, p<0.001), itching and pain (r=0.5940, p<0.001) (**Figure 4**).

The spearman heatmap showed genus *Staphylococcus, Enterobacter*, and *Streptococcus* were positively correlated with these indices, and genus *Cutibacterium, Ralstonia* etc. were negatively correlated with scar indices. (*P<0.01, **P<0.001, ***P<0.0001).

## Discussion

Recent advances have highlighted the crucial role of microbiota in the maintenance of a healthy immune system. The pathogenic bacteria invasion or colonization will cause microbiome dysbiosis, leading to inflammation and diseases. In our study, we first observed a high count of gram-positive staining in HS, and inflammation cells co-localized with the bacteria. Meanwhile, the inflammation cytokines like MCP-1, IL-6 and IL-8, and TNF-α also increased in HS. Then using 16S sequencing, we confirmed that microbiome dysbiosis occurred in HS, which was prevalent in *S.aureus.*

Traditionally, scar tissue was regarded as a “clean site” without pathogen colonization. Where are the pathogens from? A previous study revealed that most surgical site infections arouse from the skin surface, due to the microbiome community shifting from the skin surface to the deep wound (24). A study on 312 wound swab samples from 213 patients found that the most common bacterial species was *S. aureus* (37%), followed *by Pseudomonas aeruginosa* (17%), *Proteus mirabilis* (10%), *Enterobacter* spp. (6%), etc (25). Another prospective study on 1,770 wounds infection revealed that the most common causative organisms were *S. aureus* (23·7%), *Escherichia coli* (16·9%), *Staphylococcus epidermidis* (13·5%), and *Pseudomonas aeruginosa* (13·0%) (26). Similar results were reported in another study on 131 wounds, where *S. aureus*, *Pseudomonas aeruginosa*, and *Streptococci* species were the most common bacteria (27). These reports provide evidence that *S. aureus* was the major species during wound healing, which was consistent with our finding in HS. After wound healing, the tissue environment failed to remove the excess bacteria, which subsequently colonized in HS, causing microbiome dysbiosis and inducing chronic inflammation.

*S.aureus* is one of the most prevalent bacterial species causing cutaneous infections and wound inflammation (28). It is reported that *S. aureus* biofilm and toxin cause impaired granulation tissue collagen, leading to compromised wound healing (29). Moreover, previous studies have shown that *S. aureus* colonization was correlated with tissue fibrosis (30, 31). A study on bovine mammary fibrosis has found that *S. aureus* induces TLR2, TLR4, TGF-β1, and bFGF expression through AP-1 and NF-κB activation, and specific NF-κB and AP-1 inhibitors could reverse this process (32, 33). Moreover, in wound healing, *Enterobacter* could induce antibiotic resistance (34), which may be a reason for its long-term colonization and microbiota dysbiosis in scars. Furthermore, the study of intestinal fibrosis found that the inflammation caused by microbiota dysbiosis could upregulate TGFβ1, SMAD3, and α-SMA expression, and cause intestinal fibrosis (35). Thus, in our study, the higher expression of inflammation cytokines MCP-1, IL-6 and IL-8, and TNF-α may be correlated with *S. aureus* and *Enterobacter* colonization and mediate the scar formation.

It has been reported that the skin resides *Actinobacteria, Firmicutes, Proteobacteria, and Bacteroides* at the phylum level, which are essential flora to maintain skin homeostasis (17). Our study confirmed the above findings in NS and found that in HS the abundance of the phylum *Actinobacteria* decreased significantly, indicating that the loss of *Actinobacteria* may have a close association with scar progression. Studies of *Actinobacteria* in the gut have shown its role in maintaining intestinal homeostasis by secreting beneficial metabolites (36). This suggests that *Actinobacteria* may play a protective role in scar formation, being able to maintain microbial homeostasis.

Additionally, we found that at the genus level, *Staphylococcus* and *Enterobacter* in HS were strongly correlated with VSS score indices, while *Cutibacterium* and *Ralstonia* were negatively correlated with VSS score indices. Therefore, *Cutibacterium* and *Ralstonia* may be protective communities to maintain homeostasis, while *Staphylococcus* and *Enterobacter* are pathogenic communities to promote HS formation, which may be a “biomarker” of HS to predict scar formation.

Currently, there are many modalities to treat hypertrophic scars like pressure therapy, silicon, radiotherapy, cryotherapy, laser, and so on (37–41). However, the effectiveness is not satisfied. Steroid injections show better scar inhibition due to inflammation control, however, it has a high recurrence (42), because the overload bacteria were not removed or balanced. Therefore, targeting microbiome dysbiosis perhaps is a supplementary therapy for scar treatment.

How to improve microbiota dysbiosis? Currently, there are two pathways to realize it including reducing the pathogen community and increasing the protective community. In acne research, antibiotic treatment is beneficial to improve microbiome dysbiosis and recover skin health (43). In a mouse model of *E. coli*-induced prostate fibrosis, enrofloxacin treatment completely eradicated the bacteria, resolved inflammation, and attenuated collagen content (44). Another pathway to regulate microbiome dysbiosis is the use of probiotics, which consists of beneficial organisms. Nakatusji et al. found that topical application of a protective bacteria in atopic dermatitis, the colonization of *S. aureus* decreased and improved the symptoms (45). Probiotic studies have also been shown to reduce infection and enhance wound healing in burn patients (46, 47). Therefore, more studies will be performed to test these findings in HS therapy.

In summary, our study indicates that microbiome dysbiosis occurred in HS dominated by *S. aureus* colonization, which may be the causing factor of chronic inflammation. Targeting the microbiome dysbiosis is perhaps a supplementary therapy to manage scar inflammation and its formation.

## Data availability statement

The datasets presented in this study can be found in online repositories. The names of the repository/repositories and accession number(s) can be found below: https://www.ncbi.nlm.nih.gov/bioproject/PRJNA748229.

## Ethics statement

The studies involving humans were approved by the Ethics Committee of Ruijin Hospital, Shanghai Jiao Tong University School of Medicine. The studies were conducted in accordance with the local legislation and institutional requirements. Written informed consent for participation in this study was provided by the participants’ legal guardians/next of kin.

## Author contributions

XW and JY designed the research study; JY analyzed most of the data and wrote the draft of the paper; ZZ and ZM collected the hypertrophic scar and normal skin tissue from patients; JY performed most cell experiments; BY and XW revised the manuscript and contributed to the overall conclusions. All authors contributed to the article and approved the submitted version

## References

[1] Ruppert DS Mohammed MM Ibrahim MM Bachtiar EO Erning K Ansari K. Poly(lactide-co-ϵ-caprolactone) scaffold promotes equivalent tissue integration and supports skin grafts compared to a predicate collagen scaffold. Wound Repair Regen (2021) 29(6):1035–50. doi: 10.1111/wrr.12951 34129714

[2] Zhang T Wang XF Wang ZC Lou D Fang QQ Hu YY. Current potential therapeutic strategies targeting the TGF-β/Smad signaling pathway to attenuate keloid and hypertrophic scar formation. BioMed Pharmacother (2020) 129:110287. doi: 10.1016/j.biopha.2020.110287 32540643

[3] Saulis AS Mogford JH Mustoe TA. Effect of Mederma on hypertrophic scarring in the rabbit ear model. Plast Reconstr Surg (2002) 110(1):177–83; discussion 184-6. doi: 10.1097/00006534-200207000-00029 12087249

[4] Singer AJ McClain SA. Persistent wound infection delays epidermal maturation and increases scarring in thermal burns. Wound Repair Regen (2002) 10(6):372–7. doi: 10.1046/j.1524-475X.2002.10606.x 12453141

[5] Zheng J Song F Lu SL Wang XQ. Dynamic hypoxia in scar tissue during human hypertrophic scar progression. Dermatol Surg (2014) 40(5):511–8. doi: 10.1111/dsu.12474 24684437

[6] Lynam EC Xie Y Dawson R Mcgovern J Upton Z Wang X. Severe hypoxia and malnutrition collectively contribute to scar fibroblast inhibition and cell apoptosis. Wound Repair Regen (2015) 23(5):664–71. doi: 10.1111/wrr.12343 26174572

[7] Barnes LA Marshall CD Leavitt T Hu MS Moore AL Gonzalez JG. Mechanical forces in cutaneous wound healing: emerging therapies to minimize scar formation. Adv Wound Care (New Rochelle) (2018) 7(2):47–56. doi: 10.1089/wound.2016.0709 29392093 PMC5792236

[8] Hsu CK Lin HH Harn HI Hughes MW Tang MJ Yang CC. Mechanical forces in skin disorders. J Dermatol Sci (2018) 90(3):232–40. doi: 10.1016/j.jdermsci.2018.03.004 29567352

[9] Wang ZC Zhao WY Cao Y Liu YQ Sun Q Shi P. The roles of inflammation in keloid and hypertrophic scars. Front Immunol (2020) 11:603187. doi: 10.3389/fimmu.2020.603187 33343575 PMC7746641

[10] Larson BJ Longaker MT Lorenz HP. Scarless fetal wound healing: a basic science review. Plast Reconstr Surg (2010) 126(4):1172–80. doi: 10.1097/PRS.0b013e3181eae781 PMC4229131 20885241

[11] Dulay AT Buhimschi CS Zhao G Oliver EA Mbele A Jing S. Soluble TLR2 is present in human amniotic fluid and modulates the intraamniotic inflammatory response to infection. J Immunol (2009) 182(11):7244–53. doi: 10.4049/jimmunol.0803517 19454721

[12] Ogawa R Akaishi S. Endothelial dysfunction may play a key role in keloid and hypertrophic scar pathogenesis - Keloids and hypertrophic scars may be vascular disorders. Med Hypotheses (2016) 96:51–60. doi: 10.1016/j.mehy.2016.09.024 27959277

[13] Gallo RL Nakatsuji T. Microbial symbiosis with the innate immune defense system of the skin. J Invest Dermatol (2011) 131(10):1974–80. doi: 10.1038/jid.2011.182 PMC3174284 21697881

[14] Kong HH Oh J Deming C Conlan S Grice EA Beatson MA. Temporal shifts in the skin microbiome associated with disease flares and treatment in children with atopic dermatitis. Genome Res (2012) 22(5):850. doi: 10.1101/gr.131029.111 22310478 PMC3337431

[15] Minter MR Hinterleitner R Meisel M Zhang C Leone V Zhang X. Antibiotic-induced perturbations in microbial diversity during post-natal development alters amyloid pathology in an aged APPSWE/PS1ΔE9 murine model of Alzheimer’s disease. Sci Rep (2017) 7:10411. doi: 10.1038/s41598-017-11047-w 28874832 PMC5585265

[16] Zakłos-Szyda M Nowak A Pietrzyk N Podsędek A.. Viburnum opulus L. Juice phenolic compounds influence osteogenic differentiation in human osteosarcoma saos-2 cells. Int J Mol Sci (2020) 21(14):4909. doi: 10.3390/ijms21144909 32664580 PMC7404185

[17] Grice EA Segre JA. The skin microbiome. Nat Rev Microbiol (2011) 9(4):244–53. doi: 10.1038/nrmicro2537 PMC3535073 21407241

[18] Nakatsuji T Chiang HI Jiang SB Nagarajan H Zengler K Gallo RL. The microbiome extends to subepidermal compartments of normal skin. Nat Commun (2013) 4:1431. doi: 10.1038/ncomms2441 23385576 PMC3655727

[19] Ring HC Thorsen J Saunte DM Lilje B Bay L Riis PT. The follicular skin microbiome in patients with hidradenitis suppurativa and healthy controls. JAMA Dermatol (2017) 153(9):897–905. doi: 10.1001/jamadermatol.2017.0904 28538949 PMC5710430

[20] Biedermann T. Dissecting the role of infections in atopic dermatitis. Acta Derm Venereol (2006) 86(2):99–109. doi: 10.2340/00015555-0047 16648910

[21] Eichenfield LF Ellis CN Mancini AJ Paller AS Simpson EL. Atopic dermatitis: epidemiology and pathogenesis update. Semin Cutan Med Surg (2012) 31(3 Suppl):S3–5. doi: 10.1016/j.sder.2012.07.002 23021783

[22] Xi-Qiao W Ying-Kai L Chun Q Shu-Liang L. Hyperactivity of fibroblasts and functional regression of endothelial cells contribute to microvessel occlusion in hypertrophic scarring. Microvasc Res (2009) 77(2):204–11. doi: 10.1016/j.mvr.2008.08.007 18838083

[23] Guo A Zhao Z Zhang P Yang Q Li Y Wang G. Linkage between soil nutrient and microbial characteristic in an opencast mine, China. Sci Total Environ (2019) 671:905–13. doi: 10.1016/j.scitotenv.2019.03.065 30947061

[24] Wenzel RP. Surgical site infections and the microbiome: An updated perspective. Infect Control Hosp Epidemiol. (2019) 40(5):590–6. doi: 10.1017/ice.2018.363 30786943

[25] Bessa LJ Fazii P Di Giulio M Cellini L. Bacterial isolates from infected wounds and their antibiotic susceptibility pattern: some remarks about wound infection. Int Wound J (2015) 12(1):47–52. doi: 10.1111/iwj.12049 23433007 PMC7950398

[26] Twum-Danso K Grant C al-Suleiman SA Abdel-Khader S al-Awami MS al-Breiki H. Microbiology of postoperative wound infection: a prospective study of 1770 wounds. J Hosp Infect (1992) 21(1):29–37. doi: 10.1016/0195-6701(92)90151-B 1351494

[27] Haalboom M Blokhuis-Arkes M Beuk RJ Klont R Guebitz G Heinzle A van der Palen J. Wound swab and wound biopsy yield similar culture results. Wound Repair Regen (2018) 26(2):192–9. doi: 10.1111/wrr.12629 29603518

[28] Huitema L Phillips T Alexeev V Tomic-Canic M Pastar I Igoucheva O. Intracellular escape strategies of Staphylococcus aureus in persistent cutaneous infections. Exp Dermatol (2021) 30(10):1428–39. doi: 10.1111/exd.14235 PMC8110615 33179358

[29] Roy S Santra S Das A Dixith S Sinha M Ghatak S. Staphylococcus aureus biofilm infection compromises wound healing by causing deficiencies in granulation tissue collagen. Ann Surg (2020) 271(6):1174–85. doi: 10.1097/SLA.0000000000003053 PMC7065840 30614873

[30] Barbara CK. Impact of Staphylococcus aureus on the pathogenesis of chronic cystic fibrosis lung disease. Int J Med Microbiol (2010) 300(8):514–9. doi: 10.1016/j.ijmm.2010.08.002 20843739

[31] Ahmed MI Mukherjee S. Treatment for chronic methicillin-sensitive Staphylococcus aureus pulmonary infection in people with cystic fibrosis. Cochrane Database Syst Rev (2018) 2018(7):CD011581. doi: 10.1002/14651858.CD011581.pub3 PMC6513196 30052271

[32] Wu J Ding Y Wang J Wang F. Staphylococcus aureus induces TGF-β1 and bFGF expression through the activation of AP-1 and NF-κB transcription factors in bovine mammary epithelial cells. Microb Pathog (2018) 117:276–84. doi: 10.1016/j.micpath.2018.02.024 29452196

[33] Bi Y Ding Y Wu J Miao Z Wang J Wang F. Staphylococcus aureus induces mammary gland fibrosis through activating the TLR/NF-κB and TLR/AP-1 signaling pathways in mice. Microb Pathog (2020) 148:104427. doi: 10.1016/j.micpath.2020.104427 32783982

[34] Davin-Regli A Lavigne JP Pagès JM. Enterobacter spp.: update on taxonomy, clinical aspects, and emerging antimicrobial resistance. Clin Microbiol Rev (2019) 32(4):e00002–19. doi: 10.1128/CMR.00002-19 PMC6750132 31315895

[35] Zhao Z Cheng W Qu W Shao G Liu S.. Antibiotic alleviates radiation-induced intestinal injury by remodeling microbiota, reducing inflammation, and inhibiting fibrosis. ACS Omega (2020) 5(6):2967–77. doi: 10.1021/acsomega.9b03906 PMC7033964 32095719

[36] Binda C Lopetuso LR Rizzatti G Gibiino G Cennamo V Gasbarrini A. Actinobacteria: A relevant minority for the maintenance of gut homeostasis. Dig Liver Dis (2018) 50(5):421–8. doi: 10.1016/j.dld.2018.02.012 29567414

[37] So K Umraw N Scott J Campbell K Musgrave M Cartotto R. Effects of enhanced patient education on compliance with silicone gel sheeting and burn scar outcome: a randomized prospective study. J Burn Care Rehabil (2003) 24(6):411–7; discussion 410. doi: 10.1097/01.BCR.0000095516.98523.04 14610432

[38] O’Brien L Pandit A. Silicon gel sheeting for preventing and treating hypertrophic and keloid scars. Cochrane Database Syst Rev (2006) 1):CD003826. doi: 10.1002/14651858.CD003826 16437463

[39] DeBruler DM Zbinden JC Baumann ME Blackstone BN Malara MM Bailey JK. Early cessation of pressure garment therapy results in scar contraction and thickening. PloS One (2018) 13(6):e0197558. doi: 10.1371/journal.pone.0197558 29897933 PMC5999072

[40] Kuehlmann B Stern-Buchbinder Z Wan DC Friedstat JS Gurtner GC. Beneath the surface: A review of laser remodeling of hypertrophic scars and burns. Adv Wound Care (New Rochelle) (2019) 8(4):168–76. doi: 10.1089/wound.2018.0857 PMC6906753 31832273

[41] Ogawa R Tosa M Dohi T Akaishi S Kuribayashi S. Surgical excision and postoperative radiotherapy for keloids. Scars Burn Heal (2019) 5:2059513119891113. doi: 10.1177/2059513119891113 31840001 PMC6904783

[42] Morelli Coppola M Salzillo R Segreto F Persichetti P. Triamcinolone acetonide intralesional injection for the treatment of keloid scars: patient selection and perspectives. Clin Cosmet Investig Dermatol (2018) 11:387–96. doi: 10.2147/CCID.S133672 PMC6063260 30087573

[43] Park SY Kim HS Lee SH Kim S. Characterization and analysis of the skin microbiota in acne: impact of systemic antibiotics. J Clin Med (2020) 9(1):168. doi: 10.3390/jcm9010168 31936262 PMC7019264

[44] Wong L Hutson PR Bushman W. Resolution of chronic bacterial-induced prostatic inflammation reverses established fibrosis. Prostate (2015) 75(1):23–32. doi: 10.1002/pros.22886 25284058 PMC4257860

[45] Nakatsuji T Chen TH Narala S Chun KA Two AM Yun T. Antimicrobials from human skin commensal bacteria protect against Staphylococcus aureus and are deficient in atopic dermatitis. Sci Transl Med (2017) 9(378). doi: 10.1126/scitranslmed.aah4680 PMC5600545 28228596

[46] El-Ghazely MH Mahmoud WH Atia MA Eldip EM. Effect of probiotic administration in the therapy of pediatric thermal burn. Ann Burns Fire Disasters (2016) 29:268–72. PMC5346305 28289360

[47] Peral MC Martinez MA Valdez JC. Bacteriotherapy with Lactobacillus plantarum in burns. Int Wound J (2009) 6:73–81. doi: 10.1111/j.1742-481X.2008.00577.x 19291120 PMC7951207

